# Effect of COVID-19 on Male Reproductive System – A Systematic Review

**DOI:** 10.3389/fendo.2021.677701

**Published:** 2021-05-27

**Authors:** Yanfei He, Jie Wang, Junlin Ren, Yubo Zhao, Jing Chen, Xuejiao Chen

**Affiliations:** ^1^ Health Management Center, The Sixth Medical Center, Chinese PLA General Hospital, Beijing, China; ^2^ Department of Infection Control, The Sixth Medical Center, Chinese PLA General Hospital, Beijing, China; ^3^ Department of Urology, The Sixth Medical Center, Chinese PLA General Hospital, Beijing, China; ^4^ Cadre Clinic of the Sixth Medical Center, Chinese PLA General Hospital, Beijing, China; ^5^ Scientific Research and Training Office, The Sixth Medical Center, Chinese PLA General Hospital, Beijing, China

**Keywords:** COVID-19, SARS-CoV-2, semen, testis, paternal-child transmission, male reproduction, offspring, prostatic secretion

## Abstract

**Background:**

Angiotensin-converting enzyme II (ACE2), a receptor for Severe Acute Respiratory Syndrome Coronavirus 2 (SARS-CoV-2) to enter host cells, is widely expressed in testes and prostate tissues. The testis and prostate produce semen. At present, there are contradictory reports about whether SARS-CoV-2 can exist in the semen of infected men.

**Objective:**

To provide a comprehensive overview of the topic of whether COVID-19 can impact on male reproductive system.

**Methods:**

We reviewed the relevant publications on the possible impact of Coronavirus Disease 2019 (COVID-19) on male reproductive system and summarized the latest and most important research results so far. Literature published in English from December 2019 to January 31, 2021 regarding the existence of SARS-CoV-2 in semen, testis, and prostatic fluid and the effects of COVID-19 on male reproductive were included.

**Results:**

We identified 28 related studies, only one of which reported the presence of SARS-CoV-2 in semen. The study found that the semen quality of patients with moderate infection was lower than that of patients with mild infection and healthy controls. The impaired semen quality may be related to fever and inflammation. Pathological analysis of the testis/epididymis showed that SARS-CoV-2 viral particles were positive in 10 testicular samples, and the spermatogenic function of the testis was impaired. All 94 expressed prostatic secretion (EPS) samples were negative for SARS-CoV-2 RNA.

**Conclusion:**

The likelihood of SARS-CoV-2 in the semen of COVID-19 patients is very small, and semen should rarely be regarded as a carrier of SARS-CoV-2 genetic material. However, COVID-19 may cause testicular spermatogenic dysfunction *via* immune or inflammatory reactions. Long-term follow-up is needed for COVID-19 male patients and fetuses conceived during the father’s infection period.

## Introduction

COVID-19 has caused health problems in people all over the world (1). It induces respiratory-predominant multiorgan dysfunction. Sun et al. reported that viral ribonucleic acid (RNA) of coronavirus was detected in multiple organs in COVID-19 patients (2). A recent publication has detected SARS-CoV-2 in semen samples, suggesting the possibility of transmission from paternal infection to offspring (3). According to the recent bioinformatic evidence (4–6), ACE2, a target for SARS-CoV-2 infection, is predominantly enriched in the human corpus cavernosum, testis, and prostate. This raises such a question: can human sperm be a carrier for transmission of SARS-CoV-2 to the embryo, resulting in replication in the embryo, a change to the paternal genome, and thereby vertical infection of the offspring through the sperm pathway?

Salam et al. found 27 infectious viruses in human semen, including well-known viruses such as the hepatitis virus and the human immunodeficiency virus (HIV) (7). At least 11 viruses can survive in the testis and may also exist in semen, which may be related to the stable structure of the virus, specific viral epitopes, immune evasion, and other factors (7). Many viruses have been found to interact directly with sperm, impair semen quality, or impact spermatogenesis by causing local inflammation. Several opinion pieces have been published already, speculating on the possibility of testicular damage as a result of COVID-19 infection (8–10). Some authors believe that mature sperm can bind to the virus and even replicate, which increases the possibility that sperm may act as a potential vector for COVID-19 (11). On the other hand, the testis is the organ that produces sperm, and if SARS-CoV-2 infects the testis, it will affect spermatogenesis. The prostate is also one of the organs that express abundant ACE2 (12). EPS or prostatic fluid is a thin milky white fluid secreted by the prostate and is an essential component of semen. Therefore, it is reasonable to hypothesize that the prostate may be affected by SARS-CoV-2.

At the time of writing, there are contradictory reports as to whether SARS-CoV-2 can be shed into the semen of infected men. Although there is limited data, viral mRNA has been identified in the semen of COVID-19 patients, with some evidence of altered seminal parameters. This novel pandemic has forced us to consider three major issues that may affect on male reproductive system: (i) Does SARS-CoV-2 exist in seminal fluid? (ii) How does COVID-19 damage, directly and indirectly, semen or male reproduction? (iii) Can SARS-CoV-2 be transmitted to the next generation through semen? In this review, we provide a comprehensive overview of the topic of whether SARS-CoV-2 can impact on male reproductive system. We summarize the following subtopics: the existence of SARS-CoV-2 in semen, testis, and prostatic fluid; the reason why SARS-CoV-2 is positive or negative in semen; the damage mechanism and influencing factors of SARS-CoV-2 on male reproduction; and the possibility of SARS-CoV-2 impact on male reproductive system, which we believe will be helpful for future research.

## Methods

### Identify the Research Question

How does COVID-19 impact on male reproductive system directly and indirectly?

### Identify Relevant Types of Evidence

**Figure 1:**
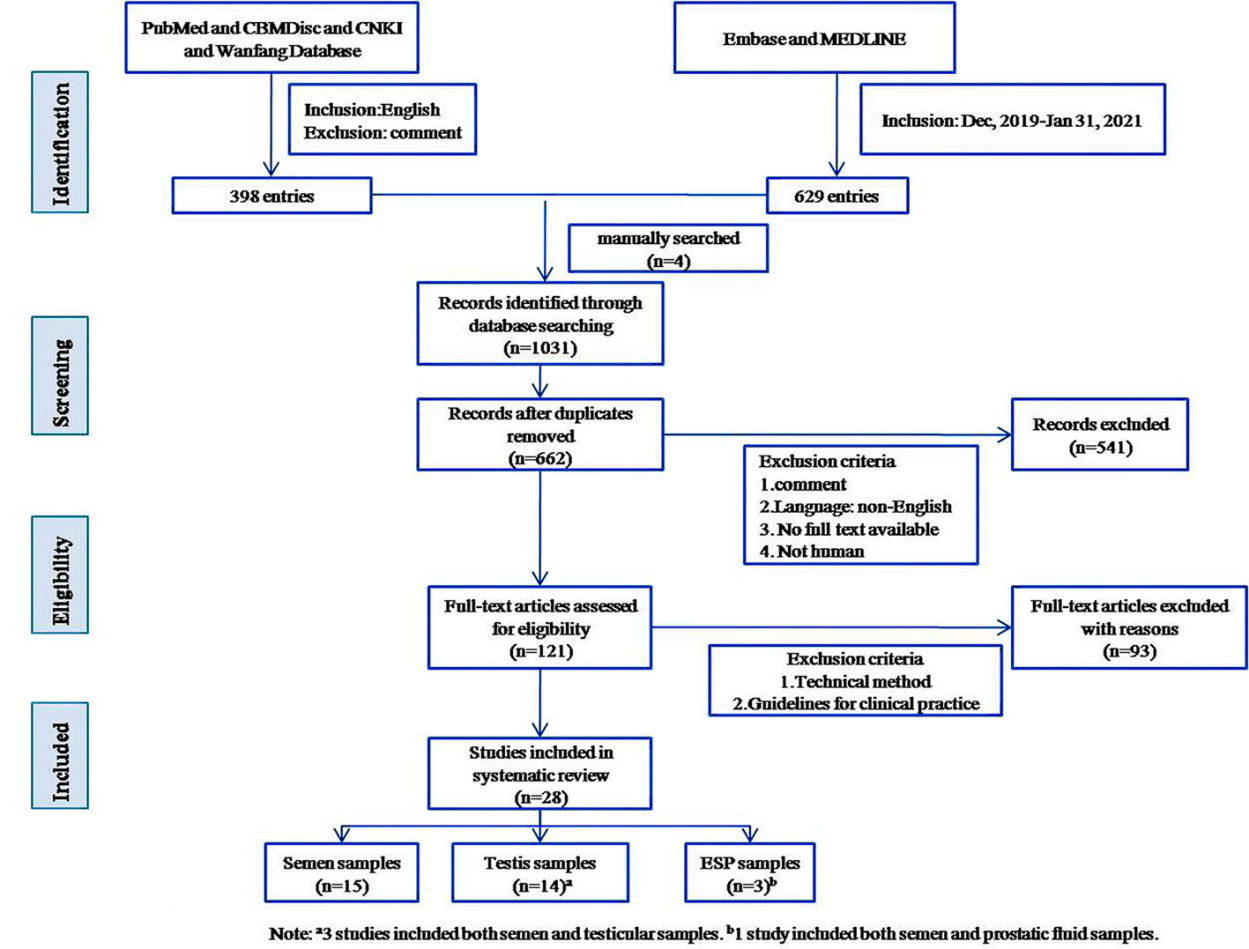
Flow diagram of literature search.

Literature published in English from December 2019 to January 31, 2021, was included in the search. We searched Pubmed, Embase, Medline, CBMDisc, CNKI, and Wanfang Database, obtaining a total of 1027 publications. Four papers were manually added, for a total of 1031. For detailed search strategies, see **Supplementary Materials 1**. We excluded redundant publications. News articles, reports, and other gray literature were included if they contained quantifiable evidence. After reading full texts and synthesizing relevant evidence, the literature was organized thematically. Themes were discussed and decided upon by all five authors, and 28 were included in the systematic review. See **Figure 1** for a visual representation of inclusion and exclusion.

### Data Charting and Summary

Two independent reviewers extracted data from the full-text papers of eligible studies, including the name of the first author, publication month and year, city and country of the study, whether patients were acutely infected or recovered, the severity of the disease of patients, age of patients, number of included patients, number of samples tested, study design, tissue assayed, main conclusion and limitations of each study, mean days until samples collection, and number of SARS-CoV-2 positive samples. A summary of the included studies is presented in **Table 1**.

**Table 1:** Characteristics of included studies.

First author [reference number]	Accessed Date	Site	Age, median (min-max), years	Stage/Severity of disease	Number of included patients (n)	Number of samples tested (n)	Tissue assayed	Study design	Main conclusion	Limitations	Mean days until semen/testicular samples collection (min-max)	Sample SARS-CoV-2 positive (n, %)
Li D (3)	May, 2020	Shangqiu, China	NP		38		Semen	Cohort study	SARS-CoV-2 can be present in the semen of patients with COVID-19.	Small sample size and the short subsequent follow-up. Lack of specific methods for the study of semen.	NP	6 (15.8)
				Recovered		23					2.5 (2-3)	2 (8.69)
				Acute		15					7.3 (2-13)	4 (26.67)
Paoli D (13)	April, 2020	Rome, Italy	31	Mild	1	1	Semen	Case report	Semen sample search for SARS-CoV-2 RNA was negative.	It cannot be ruled out whether the virus can be detected in a more severe disease case. No semen parameters measured.	8	0
Kayaaslan B (14)	August, 2020	Ankara, Turkey	33.5 (18-54)	Acute	16		Semen	cross-sectional study	SARS-CoV-2 was not detected in semen and sexual transmission *via* semen does not have an important role in the person-to-person transmission of SARS-CoV-2.	First, the study was conducted in a relatively limited number of patients and mild to moderate cases. Second, the semen parameters of the patients were not obtained.	1 (0-4)	0
				Mild		11					1 (0-7)	
				Moderate		5					1 (0-7)	
Holtmann N (15)	May, 2020	Dusseldorf, Germany		Recovered	18		Semen	Pilot cohort study	SARS-CoV-2 RNA could not be detected in semen of recovered and acute COVID-19–positive men. A mild COVID-19 infection is not likely to affect testis and epididymis function, whereas semen parameters did seem impaired after a moderate infection.	The sample size was relatively small, and there were only 2 patients with COVID-19 active infection; no sperm analysis was obtained from the individuals examined before the pandemic outbreak.	32.7 [8–54]^b^	0
			42.7 ± 10.4^a^	Mild		14					34.9 ± 11.7^a^	
			40.8 ± 8.7^a^	Moderate		4					25.5 ± 8.3^a^	
Song C (16)	April, 2020	Wuhan, China			13			cross-sectional study	2019-nCov is absent from the semen and testes in men infected by COVID-19 at both acute and recovery phases. Thus, it is highly unlikely that the 2019-nCov can be sexually transmitted by men.	Small sample size and single sampling	30 (14-42)	0
			33 (22-38)	Recovered		12	Semen				29.8 (14-42)	
			67	Deceased		1	Testis				41	
Pan F (17)	April, 2020	Wuhan, China	37 (18–57)	Recovered	34	34	Semen	Cross-sectional study	SARS-CoV-2 virus was not detected in the semen of recovered patients 1 month after diagnosis. The long-term effect of SARS-CoV-2 on male reproductive function is not clear.	Identification of SARS-CoV-2 on qRT-PCR of single ejaculated semen samples. Semen quality was not assessed.	31 [29–36]^b^	0
Ma L (18)	June, 2020	Wuhan, China	31 (25-46)	Recovered	12		Semen	Cross-sectional, pilot study	SARS-CoV-2 was undetectable in semen.	All the semen samples came from non-severe patients and most of them were in the recovery stage. The sample size is limited.	78.5 (56-109)	0
				mild		1						
				moderate		11						
Temiz MZ (19)	October, 2020	Istanbul, Turkey	37.2 (20-60)	Acute	20	20^c^	Semen	A cross-sectional, pilot study	COVID-19 has no specific deteriorative effect on male reproductive health at a short-time period.	The study has limited sample size, short follow-up time and lack of testicular histological examination.	9.5 (2-10)	0
Rawlings SA (20)	July, 2020	California, USA	38 (28-45)	Acute	6	6	Semen	Case series	SARS-CoV-2 was not present in semen.	The sample size was small, and the semen parameters of the patients were not analyzed.	12 (6-17)	0
Pavone C (21)	August, 2020	Palermo, Italy	41.1 (31-60)		9		Semen	Sampling study	Sexual transmission of SARS-CoV-2 by men recovering from mild symptoms of COVID-19 is highly unlikely.	Severe acute COVID-19 cases were not included in the selection criteria of this study	42.2 (7-88)	0
				Acute		2					10 (7-13)	
				Recovered		7					51.4 (34-88)	
Ning J (22)	April, 2020	Wuhan, China	35 (23-46)		17		Semen	Cross-sectional, pilot study	SARS-CoV-2 was not present in semen.	The sample size was small and the retrospective method was used, and the observation and follow-up time of COVID-19 patients was relatively short.	27 (12-64)	0
			38 (23-46)	Acute		9					30 (14-64)	
			30 (28-45)	Recovered		8					18.5 (12-36)	
Guo L (23)	June, 2020	Jinan, China	41 (20-62)		23		Semen	cross-sectional study	There was no SARS-CoV-2 RNA detected in semen samples, which indicates the unlikely possibility of sexual transmission through semen at about 1 month after first detection.	The sample size was small, no critical cases and no testicular biopsy. The semen parameters of the patients were not analyzed to show the abnormality of semen quality and quantity.	32 (27.5-33)	0
				Acute		12					31 (26-34)	
				Recovered		11					31 (26-34)	
Li H (24)	October, 2020	Wuhan, China	46.7 (27-83)		29				The male reproductive system could be vulnerable in COVID-19, characterized by spermatogenic dysfunction, a significant decrease in sperm count, and immune reactions in testis and epididymis.	The sample size of this study was small, and these COVID-19 patients could not be evaluated prospectively.		
			40.8 (27-53)	Recovered		23	Semen	cross-sectional study			25.8 (4-42)	0
			69.3 (51-83)	Deceased		6	Testis	Case control			NP	6
Özveri H (25)	July, 2020	Istanbul, Turkey	49	Acute	1			Case report	COVID-19 patients may show isolated genital symptoms such as testicular/spermatic cord pain and discomfort without other systemic symptoms.	Specific data for testicular biopsies and semen samples were not provided.	2	0
						1	Testis/Spermatic cord					0
						1	Semen					0
Barton LM (26)	April, 2020	Oklahoma, USA	59.5 (77-42)	Deceased	2	2	Testis	Case series	One case showed testicular atrophy and the other showed normal testis.	Small sample size and semen samples were not provided.	1	NP
Gagliardi L (27)	May, 2020	Pisa, Italy	14	Acute	1	1	Testis	Case report	In COVID-19 children could have testicular involvement, characterized by orchiepididymitis.	Specific data for testicular biopsies and semen samples were not provided.	1	NP
Bridwell RE (28)	August, 2020	Texas, USA	37	Acute	1	1	Testis	Case report	Reproductive system complications (orchitis) may be one of the characteristics of SARS-CoV-2 infection.	Specific data for testicular biopsies and semen samples were not provided.	15	NP
Marca AL (29)	July, 2020	Modena, Italy	43	Acute	1	1	Testis/Scrotum	Case report	SARS-CoV-2 infection can cause epididymitis and may have short- and long-term effects on male reproductive system.	The testes were only macroscopically examined and specific data for semen sample was not provided.	1	NP
Duarte-Neto AN (30)	May, 2020	S~ao Paulo, Brazil	69(mean)	Deceased	2	2	Testis	Case series	COVID-19 is a systemic disease that can affect the reproductive system, characterized by orchitis.	Detailed pathological results of testicular biopsy were not provided.	5	NP
Achua JK (31)	November, 2020	Florida, USA	56 (20-87)	Deceased	6	6	Testis	Case control	The histological and ultrastructural features of the testes of one patient showed COVID-19 viral particles.	The sample size was small and it was unable to assess the long-term consequences of the SARS-CoV-2 virus on spermatogenesis.	11 (2-36)	1 (16.7)
Caner E (32)	October, 2020	Istanbul, Turkey	38 (18-75)	NP	91	91	Testis	Cross-sectional, pilot study	Testicular pain was observed in COVID-19 patients, but no inflammatory markers related to predict of testicular pain or epididymal-orchitis were found.	Spermiogram and scrotal Doppler ultrasonographic evaluation could not be done for the patients.	11.8	NP
Chen L (33)	October, 2020	Wuhan, China	58.3 (43.0-73.0)	NP	142	142	Testis/Scrotum	Cross-sectional, pilot study	SARS-CoV-2 infection might specifically affect the testis, epididymis, or both.	Detailed pathological results of testicular biopsy were not provided.	15.4 (7-28)	NP
Ma X (34)	November, 2020	Wuhan, China	68.8 (51-83)	Deceased	5	5	Testis	Case control	SARS-CoV-2 could infect testicular cells through the spike glycoprotein binding mechanism.	The sample size was small, and the pre-death semen samples of the patients were not obtained.	16.2 (5-29)	2 (40)
Yang M (35)	May, 2020	Wuhan, China	65	Acute	12	12	Testis	Cross-sectional study	Testes from COVID-19 patients exhibited significant seminiferous tubular injury, reduced Leydig cells, and mild lymphocytic inflammation. Most of the testis (90%) had no evidence of SARS-CoV-2.	The sample size of this study was small, and the cases included were only in the acute stage of COVID-19.	41 (23-75)	1 (8.3)
Alkhatatbeh H (36)	July, 2020	Zarqa, Jordan	43 (1-78)		253		Testis	Descriptive study	This study did not identify any patients with COVID-19 with symptoms or signs of orchitis.	This study lacked testicular pathological examination and semen parameter analysis.	15 (9-21)	NP
				Asymptomatic		53						
				Mild		152						
				Severe		48						
Ruan Y (37)	November, 2020	Wuhan, China	31 (21-49)	Recovered	74	70^d^	Semen	Cohort study	Direct urogenital involvement was not found in the recovered COVID-19 male patients. SARS-CoV-2 RNA was undetectable in the urogenital secretions, and semen quality declined slightly.	The sample size was small and the semen parameters were lacking before infection.	80 (64-93)	0
				Mild		11						0/70^d^
				Moderate		31						
				Severe		32						
						61^d^	EPS		No SARS-CoV-2 was expressed in EPS of patients with COVID-19.			0/61^d^
Quan W (38)	March, 2020	Wuhan, Xiangyang, and Shenzhen, China	NP		23		EPS	Descriptive study	No SARS-CoV-2 was expressed in EPS of patients with COVID-19.	First, EPS were tested only in mild and common patients, there were no samples of severe patients. Second, no semen samples were obtained.	NP	0
			60.3 ± 15.3	Confirmed patients		18						
			45.6 ± 14.7	Suspected patients		5						
Zhang S (39)	June, 2020	Wuhan, China	57.5 (29-76)	Acute	10	10	EPS	Cross-sectional study	Negativity of SARS-CoV-2 in EPS and possibly exclude the sexual transmission of COVID-19.	The sample size was small and there was no semen sample.	16.4 (3-29)	0

^a^Reported as standard deviation. ^b^Reported as Interquartile range (IQR). ^c^3 patients with negative pharyngeal swabs were considered to be diagnosed with COVID-19 in this study. ^d^Only 70 semen samples and 61 prostate samples were obtained from 74 patients. EPS, expressed prostatic secretion; SARS-CoV-2, Severe acute respiratory syndrome coronavirus 2; COVID-19, Coronavirus Disease 2019; NP, Not provided; qRT-PCR, quantitative real-time polymerase chain reaction.

## Results and Analysis

**Figure 2:**
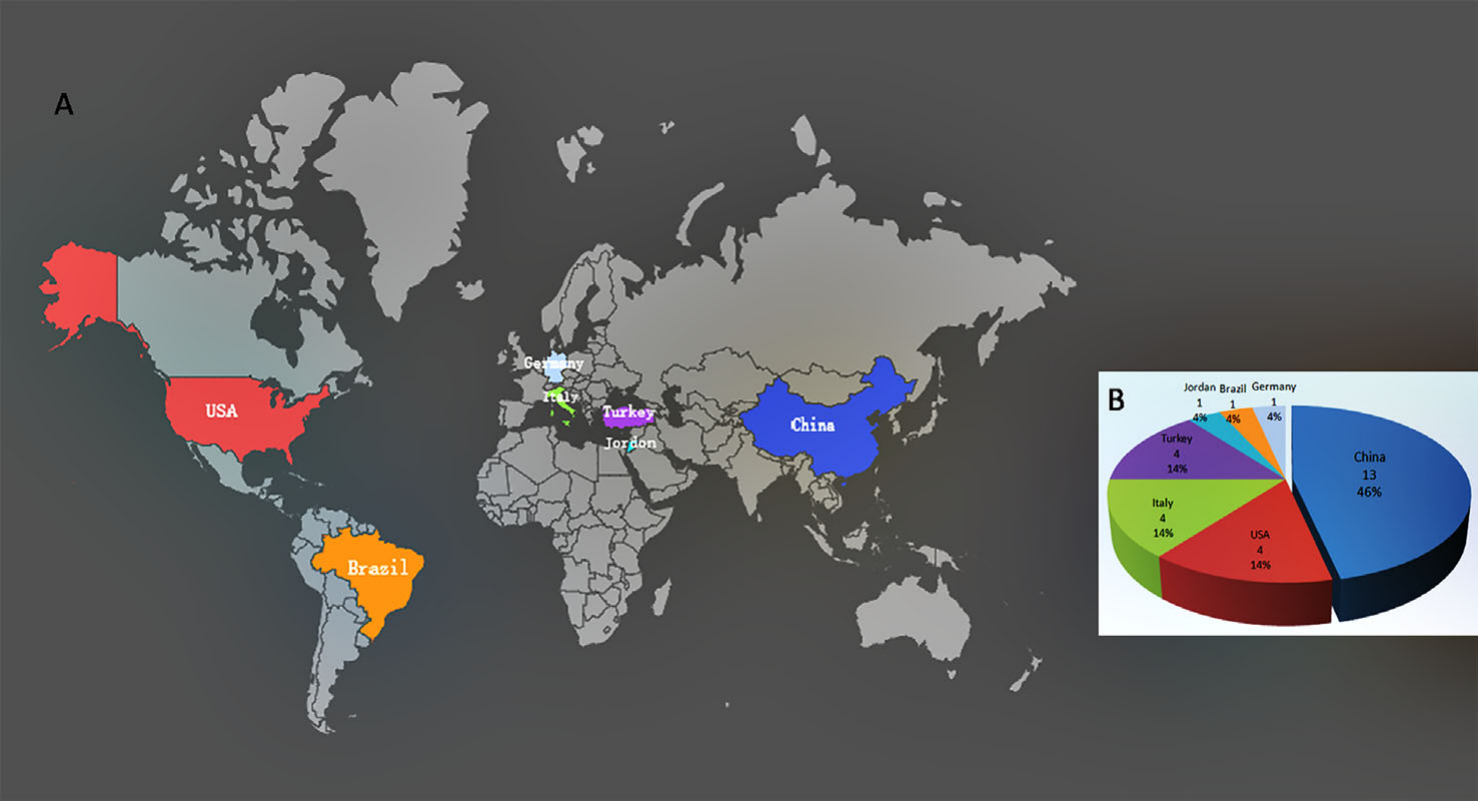
Distribution and proportion of included studies. **(A)** Distribution of 28 included studies. **(B)** Proportion of 28 included studies in seven countries.

Twenty-eight studies were eligible and included in this review (3, 13–39). They come from seven countries on four continents, including Asia (China, Turkey, and Jordan), Europe (Germany and Italy), North America (USA), and South America (Brazil), of which 13 were from China (11 from Wuhan, 39.3%) as shown in **Figure 2**. Most studies were cross-sectional. In some studies, the authors declared that they sought to fill the gaps in the literature with regards to male reproduction in the case of the COVID-19. In the 28 related studies, 15 were related to semen, 14 were related to the testis/scrotum, and three were related to the prostatic fluid. Of the 28 studies, three included both semen and testicular samples, and one included both semen and prostatic fluid samples. After reviewing the presence of SARS-CoV-2 in 15 different studies using semen samples, only one study reported the presence of the virus. Among 300 semen samples from 304 COVID-19 patients, SARS-CoV-2 RNA was detected in six (2.0%) samples, including two from convalescent males and four from acute infection. Six of the 15 studies analyzed semen parameters and found that the moderately infected patients had substandard semen quality compared with those who recovered from mild infections and healthy controls. After stratifying the patients according to the disease course, we found that the virus presented in the semen at a relatively early stage of infection, and the time interval from diagnosis to the provision of semen samples was two to 13 days. Impaired sperm quality was found in patients with moderate infection, and the decline in semen quality may be related to fever and inflammation, indicating that COVID-19 may cause spermatogenic dysfunction. Of the 14 studies on the testis/scrotum, six studies performed testis/epididymis pathological analysis in 33 deceased COVID-19 patients. Among them, 10 testicular samples were positive for SARS-CoV-2 viral particles, interstitial edema and inflammatory cell infiltration in the testis and epididymis, and obvious thinning of seminiferous tubules. Twenty-six testicular samples had impaired spermatogenesis. Scrotal discomfort of orchitis/epididymitis or spermatic cord inflammation was also reported in COVID-19 patients. In three studies on EPS, all 94 EPS samples were negative for SARS-CoV-2 RNA (see **Table 1**). All studies also show that there was no conflict of interest.

### Detection of SARS-COV-2 in Semen

Identifying the presence of SARS-CoV-2 in semen would help assess the early impact on male reproduction. Of the 15 semen studies in this review, only one showed detection of SARS-CoV-2 in semen. The research variables of semen were shown in **Table 2**.

**Table 2:** Semen Characteristics of Patients with COVID-19.

First author [reference number]	Sample size (n)	Age, median (min-max), years	Disease Stage/severity (n)			Mean days until semen sample collection (d, min-max)		Fever (n, %)	Leukocytes detected in semen (n)	Oligospermia (n)	Low sperm motility	Abnormal sperm morphology (n)	SARS-CoV−2 positive in semen sample (n,%)
Li D (3)	38	NA						NA	NA	NA	NA	NA	6 (15.8)
			Recovered		23		2.5 (2-3)						2/23
			Acute		15		7.3 (2-13)						4/15
Paoli D (13)	1	31		Mild	1		8	1 (100)	NA	NA	NA	NA	0
Kayaaslan B (14)	16	33.5 (18-54)	Acute					6 (37.5)	NA	NA	NA	NA	0
				Mild	11		1 (0-7)						
				Moderate	5		1 (0-7)						
Holtmann N (15)	18		Recovered				32.7 [8–54]^b^	10 (55.6)	14				0
		42.7 ± 10.4^a^		Mild	14		34.9 ± 11.7^a^						
		40.8 ± 8.7^a^		Moderate	4		25.5 ± 8.3^a^			c	c	NA	
Song C (16)	12	33 (22-38)	Recovered		12		29.8 (14-42)	8 (66.7)	NA	NA	NA	NA	0
Pan F (17)	34	37 (18-57)	Recovered		34		31 [29–36]^b^	NA	NA	NA	NA	NA	0
Ma L (18)	12	31 (25-46)	Recovered				78.5 (56-109)		NA	1	5	2	0
				Mild	1								
				Moderate	11								
Temiz MZ (19)	20	37.2 (20-60)	Acute		20		9.5 (2-10)	10 (50)	7	e	e	d	0
Rawlings SA (20)	6	38 (28-45)	Acute		6		12 (6-17)	6 (100)	NA	NA	NA	NA	0
Pavone C (21)	9	41.1 (31-60)			9		42.2 (7-88)	4 (44.4)	NA	NA	NA	NA	0
			Acute	2			10 (7-13)						
			Recovered	7			51.4 (34-88)						
Ning J (22)	17	35 (23-46)			17		27 (12-64)	NA	NA	NA	NA	NA	0
		38 (23-46)	Acute	9			30 (14-64)						
		30 (28-45)	Recovered	8			18.5 (12-36)						
Guo L (23)	23	41 (20-62)			23		32 (27.5-33)	7 (30.4)	NA	1	2	0	0
			Acute	12			31 (26-34)						
			Recovered	11			31 (26-34)						
Li H (24)	23	40.8 (27-53)	Recovered				25.8 (4-42)	8 (34.8)	14^d^	9^d^	NA	NA	0
				Mild	9		40.8 ± 7.2^a^						
				Moderate	14		40.9 ± 9.6^a^						
Özveri, H (25)	1	49	Acute		1		25	NA	NA	NA	NA	NA	0
Ruan Y (37)	70^f^	31 (21-49)	Recovered		74		80 (64-93)	56 (80)	NA	d	d	e	0
			Mild	11									
			Moderate	31									
			Severe	32									

^a^Reported as standard deviation. ^b^Reported as Interquartile range (IQR). ^c^Patients with a moderate infection have a statistically significant impairment of sperm quality (sperm concentration, total number of sperm per ejaculate, total number of progressive motility, total number of complete motility) compared with men recovered from a mild infection and the healthy control group. ^d^There was a significant difference between the observation group and the healthy control group. ^e^There was no significant difference between the patients and the healthy control group. ^f^Only 70 semen samples were obtained from 74 patients. NA, Not applicable.

#### Positive Results and Interpretation

Li et al. (3) detected SARS-CoV-2 qualitative real-time polymerase chain reaction (RT-PCR) positive in the semen of six out of 38 patients (15.8%). Of six patients, two were in the recovery stage (two of 23 cases), and four were in the acute stage (four of 15 cases). To our knowledge, this is the only study reporting a positive result in semen. The study pointed out that the interval times from onset of symptoms to the testing of semen were 2.5 days (2-13 days) and 7.3 days (2-13 days), respectively. This was a shorter duration than most studies (13, 15–24, 37) that reported negative viral RNA in semen but a longer duration than one study (14) and one case report (25) that reported negative viral RNA within an interval time of 0-7 days (median: one day) and two days, respectively, suggesting that SARS-CoV-2 perhaps shed into semen at a relatively early stage of COVID-19. Some authors also believe that the presence of SARS-CoV-2 in semen is a consequence of the residual urine shedding, as the urinary and genital tract are overlapped in males at the distal end (40). In this study, semen samples were tested for SARS-CoV-2 by RT-PCR, but neither the limit of detection (LoD) nor the cycle threshold (Ct) values were described. Although the RT-PCR is with high specificity, many factors can cause false positive results (41). Moreover, the semen collection modality is not provided (masturbation, electrovibrator, or other), as in the process of sample collection, if the samples are not collected according to aseptic conditions, a possible contamination with RNA fragments from hands or respiratory droplets may lead to false positive results. Moreover, a ‘positive’ RT-PCR result reflects only the detection of viral RNA and does not necessarily indicate the presence of viable virus (42). There are still many questions to be answered as to whether it can impact on male reproductive system, such as (i) whether the virus binds to sperm or even internalizes in semen? (ii) Is it capable of active replication and potential infection? (iii) For how long does it remain detectable in semen?

### Negative Results and Interpretation

In this review of 15 semen samples, 266 subjects (262 semen samples) from another 14 studies indicated that SARS-CoV-2 was not detected in semen (13–25, 37). The protection of the blood-testis barrier and the low level of viremia in COVID-19 patients are both possible reasons (43). Besides, since most of the semen specimens in these studies were obtained from patients in the recovery stage, the virus (if it ever existed in semen) may have been cleared up by the detection time. In the previous studies (13, 44, 45), viremia and viruria were reported to be very low and transient. Therefore, the negative results can be interpreted as the viral level in the patient matching the course of the patient’s progressive clinical recovery (46).

Six of the above studies (15, 18, 19, 23, 24, 37) further investigated the semen parameters (**Table 2**). Holtmann et al.’s study (15) showed that although SARS-CoV-2 RNA was not detected in the semen samples of recovered or acutely infected patients, patients with a moderate infection have statistically significant impairment of sperm quality (sperm concentration, total number of sperm per ejaculate, total number of progressive motility, total number of complete motility) compared to patients who recovered from a mild infection and the healthy control group. Ma et al. (18) studied the semen samples of 12 male patients with COVID-19: five patients had low sperm motility (defined as progressive spermatozoa (PR) + non-progressive (NP) <40%), two patients had poor sperm morphology (defined as normal morphology <4%), one patient was diagnosed with oligozoospermia (defined as <20 million/mL) before COVID-19 and did not have much alteration in his semen after SARS-CoV-2 infection. Guo et al. (23) investigated the semen quality of 23 male patients with COVID-19: one patient was diagnosed with oligozoospermia, two patients had low sperm motility. Ruan et al. (37) conducted a study on 70 semen samples from 74 COVID-19 recovered patients and found that the overall semen quality of COVID-19 recovered patients was higher than the lower reference limit issued by the World Health Organization and compared to the healthy control group, the sperm density, total sperm count, and total motility decreased significantly. Li et al. (24) reported that in the semen samples of 23 COVID-19 convalescent patients, 14 (60.9%) patients had high leukocyte count, proinflammatory cytokines and chemokines, including Interleukin-6 (IL-6), tumor necrosis factor-α (TNF-α), and monocyte chemotactic protein-1 (MCP-1), which were higher than those of control participants. An increased concentration of seminal leukocytes may cause sperm abnormalities by activating reactive oxygen species. When male individuals are infected with SARS-CoV-2, changes in the ACE2 signaling pathway, oxidative stress, and inflammation may cause sperm DNA breakage and an increase of sperm DNA fragmentation index (DFI) (47, 48). When the DFIs exceed 25%, the DNA carried by spermatozoa may be accompanied by gene deletion, which can lead to miscarriage, malformation, and fetal arrest after conception (49).

In Holtmann et al.’s study (15), when the recovered patients were stratified by whether fever occurred during infection, the fever-positive group (N=10) showed significantly lower sperm concentration and complete motility than the fever-negative group (N=8). Temiz et al. also found that the percentage of normal sperm morphology in the observation group was significantly lower than that in the healthy control group (19). The authors attributed this finding to the fever seen in all COVID-19 patients in the observation group. In the cohort study of Ruan et al. (37), 56 of the 74 COVID-19 recovered patients had a fever. Compared to the healthy control group, the sperm density, total sperm count, and total motility of 70 semen samples in the cohort decreased significantly. Carlson et al. (50) and Andrade-Rocha FT (51) investigated the effects of fever on semen parameters and their results supported this thought.

Holtmann et al. (15) found that after stratification according to the severity of the disease, patients who had recovered from moderate disease (N=4) showed significantly lower sperm quality (i.e., sperm concentration, total number of spermatozoa per ejaculate, total number of spermatozoa with progressive motility and total number of completely motile spermatozoa) than those who recovered from a mild infection (N=14) or healthy controls (N=14). These results suggest that COVID-19 impairs semen quality in a disease severity-related manner.

SARS-CoV-2 RNA was undetectable in semen samples collected from most of the convalescent and acute patients with COVID-19, indicating that the existing data did not support the existence of SARS-CoV-2 in semen and the risk of damage to the embryo *via* the sperm route is extremely low. However, the sperm quality was found to be impaired in patients with moderate infection, confirming that COVID-19 can cause a decrease in semen quality, and suggesting that the decline in semen quality may be related to fever and the severity of the disease. Of course, some limitations should be taken into account in the interpretation of the above results. First, due to the limited sample size and only a single semen sample collection performed after infection, not all studies have detected semen parameters. Among the semen samples that have been evaluated, there is significant biological variation in semen parameters. Therefore, a further well-designed prospective cohort study is needed to analyze a series of samples from the onset of symptoms to complete recovery to clarify the effect of COVID-19 on spermatogenesis. Secondly, all the semen samples came from non-critically ill patients, and most of them were in the recovery stage, so we cannot rule out the existence of SARS-CoV-2 in the semen of more seriously ill patients. Finally, our preliminary results are short-term data as we lack data on the long-term effects of SARS-CoV-2 on male reproduction.

### Testis/Epididymis or Scrotum Research Results and Interpretation

In this review, there are 14 studies on the testis/epididymis or scrotum, with a total of 333 patients (**Table 3**). The nasopharyngeal/oropharyngeal swabs of all patients were positive for SARS-CoV-2. In six of the 14 studies on the testis/scrotum abovementioned (24, 26, 30, 31, 34, 35), testicular/epididymal pathological analysis was performed on 33 deceased COVID-19 patients, and in 10 of 33 testicular samples positive for SARS-CoV-2, virus particles were identified.

**Table 3:** Descriptive Results in the Testes/Scrotum of Patients With COVID-19.

First author [reference number]	Sample size (N)	Age, median (min-max), years	Disease stage/severity	Confirmed disease duration (d)	Hospital stay (d)	Comorbidity	Fever (n, %)	Testicular/Scrotal symptoms	Cause of death	Ultrasonography	CT	Urine microbiological investigations	Pathological investigation	Nasopharyngeal/Oropharyngeal swab qRT-PCR	Testicular sample qRT-PCR positive (n, %)
Song C (16)	1	67	Acute	41	41	No comorbidity	1 (100)	NA	COVID-19	NA	Chest CT were patchy shadowing	NA	NA	Positive	NA
Li H (24)	6	69.3 (51-83)	Deceased	14.1 (0-23)	17 (5-21)	Hypertension	3 (50)	NA	COVID-19	NA	NA	NA	Interstitial edema and congestion were both in testes and epididymides, the proportion of apoptotic cells, T-lymphocyte (CD3+) and macrophage (CD68+) infiltration in the testes of COVID-19 deceased patients was significantly increased, IgG was presented within seminiferous tubules and seminiferous tubules became thinner.^a^	Positive	6 (100)
Özveri H (25)	1	49	Acute	2	25	Hyperlipidemia and insulin resistance	NA	Swelling and pain	Recovered	Spermatic cord inflammation with no orchitis	Normal	Negative	NA	Positive	0
Barton LM (26)	2	59.5 (77-42)	Deceased	1	1	Hypertension, remote deep vein thrombosis, myotonic muscular dystrophy	2 (100)	NA	COVID-19	NA	Chest CT showed bilateral ground-glass opacities	NA	The 42-year old patient showed testicular atrophy and the 77-year old patient showed normal testis.	Positive	NA
Gagliardi L (27)	1	14	Acute	1	8	NA	1 (100)	Swelling and pain	Recovered	Inflammation of the epididymis with reactive hydrocele	NA	Negative	NA	Positive	NA
Bridwell RE (28)	1	37	Acute	15	2	No comorbidity	1 (100)	Swelling and pain	Recovered	Bilateral non-specific increased blood flow was present consistent with orchitis.	Peripheral mid to lower hazy pulmonary opacities	Negative	NA	Positive	NA
Marca AL (29)	1	43	Acute	1	4	Type 1 diabetes	1 (100)	Severe bilateral testicular pain	Irreversible cardiogenic shock	Left epididymis with mild accentuation of the vascularization pattern, epididymitis.	Multiple thickening concomitant with bilateral consolidations.	Negative	NA	Positive	NA
Duarte-Neto AN (30)	2	69(mean)	Deceased	2	5	Systemic arterial hypertension, diabetes mellitus, Chronic cardiopathy	2 (100)	NA	COVID-19	NA	NA	NA	Orchitis.	Positive	NA
Achua JK (31)	6	56(20-87)	Deceased	11 (2-36)	2 (0-6)	Type 2 diabetes meletus, cardiovascular disease, hypertension	NA	NA	COVID-19	NA	NA	NA	Three patients had impaired spermatogenesis, interstitial macrophage and leukocyte infiltration.	Positive	1 (16.6)
Caner E (32)	10	46	Acute	10	NA	NA	NA	Swelling and pain	Recovered	NA	NA	NA	NA	Positive	NA
Chen L (33)	32	70.3	Acute	14.6 (9-27)	NA	Chronic kidney disease, diabetes, Coronary heart disease	13	Swelling and pain	b	Acute orchitis, epididymitis, or epididymo-orchitis	Changes characteristic of viral pneumonia	NA	NA	Positive	NA
Ma X (34)	5	68.8 (51-83)	Deceased	5	16.2 (5-29)	Hypertension, Coronary disease	NA	NA	COVID-19	NA	Bilateralground-glassopacities	NA	Numerous degenerated germ cells had sloughed into the lumen of seminiferous tubules, the number of germ cell marker positive cells was dramatically reduced.^f^	Positive	2 (40)
Yang M (35)	12	65	Acute	42 (23-75)	NA	Hypertension, chronic renal disease, coronary heart disease8	10 (83.3)	NA	COVID-19	NA	NA	NA	Sertoli cells showed swelling, vacuolation and cytoplasmic rarefaction, detachment from tubular basement membranes, and loss and sloughing into lumens of the intratubular cell mass. Interstitial edema, mild inflammatory infiltration, composed of T lymphocytes and histiocytes.	Positive	1 (8.3)
Alkhatatbeh H (36)	253	43 (1-78)	Acute	15 (9-21)	NA	NA	200^c^ (79.1)	No testicular or scrotal symptoms	Recovered^d^	No testicular or scrotal symptoms	Radiographic evidence of pneumonia ^e^	Negative	NA	Positive	NA

^a^Compared with age-matched prostate cancer patients. ^b^The author did not specify the number of patients who recovered or died in these 32 cases. ^c^153 patients with mild symptoms. ^d^Five (2%) patients died of COVID-19. ^e^200 patients had CT examination. ^f^Compared with age-matched non-infected patients. CT, Chest computed tomography; COVID-19, Coronavirus Disease 2019; qRT-PCR, quantitative real-time polymerase chain reaction; NA, Not applicable.

Yang et al. (35) performed an autopsy on the testes of 12 COVID-19 patients and found that germ cells were extensively damaged, which were characterized by Sertoli cell swelling, vacuolization, cytoplasmic rarefaction, detachment from the tubular basement membrane, thinning of seminiferous epithelium, leukocyte infiltration, and significant increase of CD3+ T lymphocytes and CD68+ macrophages in the testicular interstitium. SARS-CoV-2 was not detected in 11 of the 12 testicular tissues, Only one of the cases was positive for SARS-CoV-2 in testicular tissue. The lung, kidney, and spleen tissues of the patient were positive for SARS-CoV-2 by RT-PCR, indicating that the patient had a high viral load and RT-PCR was likely to detect the virus present in the blood but not in the testicular tissue. Examination of the testicular and epididymal tissues of six deceased COVID-19 patients revealed the presence of interstitial edema, congestion, and red blood cell exudation (24). A significant increase in apoptotic cells within seminiferous tubules and an increased infiltration of CD3+ T lymphocytes and CD68+ macrophages in testicular interstitium were observed (24). The deposition of IgG’s in the seminiferous epithelium was observed as well as the thinning of seminiferous epithelium (The control group was defined as age-matched prostate cancer patients without COVID-19).

Ma et al. (34) performed an autopsy on the testes of five deceased COVID-19 patients. They found that in all five COVID-19 patients, numerous degenerated germ cells had sloughed into the lumen of seminiferous tubules. In four of the five cases, germ cell loss was massive, the number of DDX4 (a germ cell marker)-positive cells was dramatically reduced in all testicular specimens (The control group was defined as age-matched patients without COVID-19).

In the study conducted by Achua et al. (31), hematoxylin and eosin (H&E) histomorphology showed that three of the six COVID-19 biopsies had normal spermatogenesis while the remaining three had impaired spermatogenesis. Transmission electron microscopy (TEM) showed SARS-CoV-2 in the testis tissue of one COVID-19 patient autopsy. An H&E stain on the same autopsy case demonstrated interstitial macrophage and leukocyte infiltration. Immunofluorescent stained slides from six COVID-19 men demonstrated a direct association between increased quantitative ACE-2 levels and spermatogenesis impairment.

The above studies suggest that COVID-19 may damage male testes and lead to impaired spermatogenesis. Barton et al. (26) reported that a 77-year-old male who died six days after the onset of symptoms displayed pathologically normal testes, while another 44-year-old patient had testicular atrophy, also suggesting that SARS-CoV-2 infection may damage the testis.

In addition, two studies (32, 33) reported scrotal discomfort in 42 COVID-19 patients during the acute phase of infection, including scrotal pain, swelling, or testicular pain. Chen et al. (33) observed that men with severe COVID-19 had a significantly higher possibility of epididymo-orchitis compared to the non-severe COVID-19 group. Four case reports (25, 27–29) of the 14 studies also indicated that COVID-19 patients had testicular pain or external genital pain as the first clinical sign of COVID-19. They were aged 14-49 years old and were diagnosed with SARS-CoV-2 infection, while urine microbiological culture for other potential urinary tract infections were ruled out, which suggested that their symptoms could have been caused by COVID-19. However, there were also reports of different results. Alkhatatbeh et al. (36) followed up 253 COVID-19 male patients with a mean (range) age 43 (1-78) years until their recovery or discharge (5-21 days), and they did not observe any symptoms or signs of orchitis. The difference between this conclusion and the abovementioned conclusion may be related to the mild condition of the patients (205 asymptomatic or mildly symptomatic patients, 205/253) and the short follow-up period (the longest follow-up time was 21 days).

To date, studies analyzing SARS-CoV-2 in testicular biopsies have lacked appropriate controls, and most of the participants have suffered from predominantly mild infections and have been tested shortly after the infection, thereby increasing the complexity of interpreting the results. It is necessary to conduct long-term follow-ups of the reproductive function of recovered young male patients and to further study this particular clinical sign and symptom of testicular or scrotal involvement to determine the pathophysiology along with the reproductive sequelae in men and develop standard therapeutic strategies to aid these patients. The studies above do have some limitations. First, in nine of the 14 studies on the testis or scrotum, 76 patients/samples did not have a comprehensive genitourinary examination, only six studies out of 33 patients/samples had histopathological evidence to prove whether there were SARS-CoV-2 viral particles in the testis or epididymis of COVID-19 patients, while the pathological features of other patients were unclear, which limited the explanation regarding orchitis or scrotal symptoms during SARS-CoV-2 infection. We cannot state with certainty that SARS-CoV-2 was responsible, because genitourinary tract infections may have resulted in orchitis or scrotal pain and swelling as well. Secondly, the observation or follow-up time on COVID-19 patients is short, and the incubation period of testicular involvement or the long-term effects on spermatogenic function remains unknown. Thirdly, most patients were asymptomatic or had mild-to-moderate symptoms, and it was possible that the viral threshold was not achieved to cross the blood-testis barrier (BTB), as some researchers suggested that higher viral loads are associated with more severe symptoms with more extrapulmonary involvement  (52).

### EPS Examination Results and Interpretation

In three studies on EPS (37–39), 89 EPS samples were from patients with a positive nasopharyngeal swab test, five samples were from suspicious patients, and all 94 EPS samples were negative for SARS-CoV-2 RNA.

The EPS is secreted by the prostate and is an essential component of semen, accounting for one-tenth to one-third of the volume of the ejaculation. The prostate fluid protects and nourishes the sperm cells. During ejaculation, the muscles of the gland help in pushing the seminal fluid through the urethra during ejaculation. If receptors for SARS-CoV-2 are present at different stages of the male reproductive system, it is not unreasonable to think that the virus could be found in semen either by alteration of the BTB or by excretion into the prostatic fluid. ACE2 and transmembrane serine protease 2 (TMPRSS2) are highly expressed by the epithelium of the human prostate (53). Therefore, it is reasonable to hypothesize that the prostate may be affected by SARS-CoV-2. If this hypothesis could be confirmed, it would provide an explanation for the virus’s presence in the seminal fluid and could justify the persistence of viral RNA in recovering patients. According to the current research results, EPS is negative for SARS-CoV-2 RNA, indicating that the virus may not exist in EPS, suggesting that SARS-CoV-2 does not have an important role in affecting sperm *via* prostatic fluid during COVID-19’s period. This result further supports that there is little possibility of SARS-CoV-2 in the semen of COVID-19 patients. However, these results must be interpreted cautiously due to some limitations. First, the sample size was small: only three studies with a total of 94 samples. And there was a lack of EPS sample test for severely and critically ill patients. Additionally, two of these studies did not obtain semen samples simultaneously, lacking the correlation between EPS characteristics and semen characteristics during the course of COVID-19.

## Discussion

The impact of COVID-19 on male reproductive system may involve multiple possible mechanisms: (i) The combination of SARS-CoV-2 and ACE2 may directly impair sperm or testicular function. (ii) The damage to the male reproductive system caused by COVID-19 immune response. (iii) The effect of hormone level on male fertility.

The effect of SARS-CoV-2 on the spermatozoal genetic material includes the direct damage to sperm and the indirect damage of the inflammatory response induced by the virus. Male reproductive impact on offspring can be reflected in the decrease of semen and sperm parameters, as well as the weakening of sperm function. If SARS-CoV-2 can replicate in sperm like hepatitis B virus (54), its genetic material can be transmitted from father to son. To date, studies have proved that spermatozoa have all of the machinery needed to bind this virus, fuse with it, and even achieve reverse transcription of the viral RNA into proviral DNA (11). SARS-CoV-2 mediates sperm chromatin and DNA damage, which will cause changes in the paternal genomes and may eventually lead to abnormal outcomes of offspring such as abortion and malformation.

SARS-CoV-2 is a novel β-coronavirus, its genome encodes four main structural proteins: Spike (S), envelope (E), membrane (M), and nucleocapsid (N) (55). SARS-CoV-2’s 3D (three dimensional) structure increases its binding affinity for ACE2 that serves as a viral receptor (56). Also, a newly-discovered poly alkali (furan) cleavage site of SARS-CoV-2 increases the virus’ ability to internalize into cells (4). Recently (57) it has also been reported that SARS-CoV-2 creates a novel invasion route by binding S protein to BSG through which it invades host cells.

Sperm cells hold all of the ACEs, including ACE2, as well as TMPRSS2, which can enhance ACE2-mediated viral entry (58–62). The spike protein on SARS-CoV-2 specifically targets ACE2, removes phosphoinositide 3-kinase and protein kinase B (PI3K/AKT), and dephosphorylates it, causing sperm apoptosis, thereby reducing sperm viability (63). In the above four studies (15, 18, 23, 37), the total sperm count and total motility of COVID-19 patients decreased significantly, which may be associated with SARS-CoV-2 virus-mediated direct damage to sperm. Angiotensin II also further affects sperm fertilization and motility by stimulating angiotensin II type 1 receptor (AT1R) and angiotensin II type 2 receptor (AT2R) (64–66), and also stimulates the acrosome reaction of sperm cells and makes them exposed to high levels of Ang II for a long time, leading to apoptosis and cell senescence.

The testes are mainly constructed by seminiferous tubules and intertubular tissue. The seminiferous tubules are the places where the sperm are generated. They are composed of sperm-producing cells (spermatogonia) and the supporting Sertoli cells. ACE2 is highly expressed in testicular cells, mainly distributed in spermatogonia, Leydig, and Sertoli cells (4, 5, 67), which indicates that the testis could be a potential target for SARS-CoV-2. If the virus causes damage to these cells, the spermatogenesis process will be affected. Besides, ACE2 receptor sites in testicular tissue have the capability to harbor the SARS-CoV-2 virus, with eventual shedding into the semen. This can theoretically explain the testicular damage after SARS-CoV-2infection. Therefore, the subsequent impact of SARS-CoV-2 on male reproductive risk and embryonic development is gaining interest among scientists. A study performed following the outbreak of SARS-COV infection in 2002 showed that orchitis was a recognized complication of SARS (68). The BTB is a physical barrier between seminiferous tubules and capillary blood, which can prevent viruses, toxins, and immune cells from entering seminiferous tubules. However, some viruses, such as Ebola virus, human immunodeficiency virus (HIV), and Zika virus, have been confirmed to traverse the BTB and elicit an immune response in the testis (7, 69). Similarly, some authors believe that even with the presence of the blood-testis barrier and immune privilege, SARS-CoV-2 with a size of 70-100 nm may cross the blood-testis barrier, triggering an immune response in the testis and leading to autoimmune orchitis (17).

Fever is one of the common symptoms of COVID-19, and the increased body temperature can hinder spermatogenesis. Studies have shown that the survival temperature of sperm cannot exceed 35°C, which is 2°C lower than the normal body temperature. If the fever exceeds 38.5°C, it will affect the quality of sperm for up to three months (70). As a result, even a fever with a limited duration can decrease sperm quality, which may lead to birth defects in offspring. Xu et al. (71) reported that meiotic germ cell apoptosis is related to high temperature. Ma L and Temiz MZ (18, 19) described a significant decrease in the percentage of normal morphology in the semen analysis of the COVID-19 patients. The authors attributed this finding to the fever seen in all COVID-19 patients, which was described and supported previously in the literature (50, 51).

The results of viral orchitis observed in the autopsy specimens (24, 31, 34, 35) were mainly characterized by testicular seminiferous tubule injury, interstitial edema dominated by T lymphocytes, and mild lymphocytic inflammation. These pathological findings suggest that alternations in cytokine levels may damage spermatogenesis (72, 73). SARS-CoV-2 may impair the spermatogenic function of testis in male COVID-19 patients *via* immune response, which further impacts the health of offspring. Interestingly, most of the studies included in our review have not detected viral genomic materials in the testicular tissue specimens (**Table 3**), which may only explain that testes are unlikely to be direct damage by the virus but cannot deny the objective fact of testicular damage. The testicular impairment may be attributed to secondary inflammatory and immunological reactions (74, 75), hyperthermia, hypoxia, and steroid drugs. Studies have shown that persistent high body temperatures during viral infections may tamper with the BTB (76). Even mild scrotal heat stress may lead to BTB leakages and allow for the passage of macromolecular substances to the testis.

The autoimmune response induced by SARS-CoV-2 may also be one of the damage factors on male reproductive system. Li H and Yang M (24, 35) analyzed the pathological changes of the testes from deceased COVID-19 patients. Immunohistochemistry results showed abundant IgG precipitation in the seminiferous epithelium of SARS testis, suggesting immune response as the cause of the injury. These inflammatory cells can activate an autoimmune response to destroy the seminiferous epithelium directly and decrease the semen quality. Several studies have shown that male infection with SARS-CoV-2 may lead to acute hypogonadism, which is related to the increase of proinflammatory cytokines, mainly IL-1β, IL-6, and TNF-α (77–79). Renu et al. also believe that it is also closely associated with interferon-γ and mediated IL-4 (80).

SAR-CoV-2 can also operate *via* multiple possible mechanisms which may lead to disruption of male reproductive functions and cause potential damage to offspring development. SAR-CoV-2 activates oxidant-sensitive pathways *via* inflammatory responses, thereby inducing oxidative stress (OS). OS can cause peroxidative damage to the sperm plasma membrane and destroy the integrity of DNA in the sperm nucleus. It leads to a decrease in sperm count and an increase in abortion rate (81). In addition to SARS-CoV-2 infection, antiviral drugs, like ribavirin, can reduce the testosterone level, leading to spermatogenesis disorders and sperm deformities, which are also associated with the induction of OS (82, 83). Moreover, COVID-19 causes psychological stress to induce systemic OS (84), synthesis and release high levels of nitric oxide (NO), thereby affecting androgen secretion, impairing spermatogenesis, inhibiting sperm motility, and causing adverse consequences to offspring (85).

Semen parameters are indeed a direct reflection of affecting offspring, but the alternations of hormone levels also provide indirect clinical evidence for SARS-CoV-2 attacking testes. SARS-CoV-2 infection may lead to male hypogonadism in the acute stage (86). Both androgen receptor and ACE2 gene loci are on the chromosome X. The genetic polymorphism of this chromosome and the subsequent increase in endogenous androgen action may explain the higher susceptibility of men to SARS-CoV-2 infection (87). A recent study compared 81 COVID-19 patients of childbearing age with 100 healthy age-matched men. It was found that the serum luteinizing hormone (LH) level in the observation group was significantly higher, while the ratio of testosterone to LH (T/LH) was significantly lower than that in the control group (88). High levels of inflammatory cytokines in COVID-19 patients, such as IFN-γ and IL-2, can stimulate lipid peroxidation of human spermatozoa and increase DNA fragmentation of spermatozoa (89), thus impairing sperm motility and viability (90). These findings suggest that the virus may play an indirect role by changing the hypothalamic-pituitary-testicular (HPT) axis. Yet another study showed that male patients had increased levels of LH and follicle-stimulating hormone (FSH) with decreased levels of testosterone. The authors believe that the testosterone level of COVID-19 patients is decreased by secondary immune response, that is, SARS-CoV-2 infection down-regulates HPT-independent testosterone (91). However, data on the dynamic regulation of cortisol mediated by COVID-19 is not available yet. It is unknown whether the low testosterone levels observed in COVID-19 patients are the cause or result of severe infection (92, 93). Therefore, it is necessary to further study the relationship between testosterone level and SARS-CoV-2 infection to explore the damage of COVID-19 to male reproduction and embryonic development from the hormone level.

As far as we know, our review is the first comprehensive description of the semen characteristics and testicular and prostate involvement of male COVID-19 patients. So far, no definitive data has been obtained to track the reproductive functions in men recovered from COVID-19. Therefore, our review can provide references for future research on evidence-based medicine. This review also has certain limitations. First, most of the studies included in this review are limited by a small sample size and short follow-up period (the longest follow-up time is 109 days), while the effect of SARS-CoV-2 on sperm requires long-term follow-up. Therefore, detailed information on virus shedding, survival time, and concentration in semen requires larger and longer cohort studies of currently infected subjects. Second, most of the studies were conducted in mild to moderate cases. They lacked data on severe and critical cases and could not investigate the correlation between severity, viral load, course of the disease, and semen outcome. Third, to date, all studies lack semen analysis before SARS-CoV-2 infection and only perform semen analysis once after infection. They lacked adequate and appropriate controls, which limits the characterization of data on possible early viral shedding in semen. Prior studies have suggested that higher viral loads are associated with more severe disease symptoms. These preliminary data cannot characterize the long-term impact of COVID-19 on offspring. Fourth, the effects of drugs used to treat COVID-19 on semen, testicles, and prostate are not considered. These drugs can possibly affect the viral load and sperm parameters in the body. Finally, because of the exponentially increasing number of publications on SARS-CoV-2, there may be a risk of bias toward reporting positive outcomes. It is necessary to carefully interpret whether the results of the 28 studies extracted by our review have regional or population bias (**Figure 2**) and whether they can represent all populations.

## Conclusion

Collectively, the influence of SARS-CoV-2 infection on male reproductive system is a novel research field. We can draw several conclusions by reviewing these studies. First, they provide direct evidence that COVID-19 may induce orchitis *via* immune or inflammatory reactions, potentially damage spermatogenesis, decrease sperm quality in moderately infected patients, and further adversely affect male reproduction. Secondly, based on the current preliminary understanding, although SARS-CoV-2 shedding into semen or being present in the testes still lacks good evidence, these studies add more evidence that SARS-CoV-2 is unlikely to be present in the semen of patients with mild to moderate symptoms or recovery from COVID-19, and the risk of impact on male reproductive system is minimal. COVID-19 causes multi-organ damage, including the reproductive system, which raises concern as to the consequences of paternal reproduction for the future. We still have a lot of related research to do: (i) Is SARS-CoV-2 present in semen during different phases of patient infection? If SARS-CoV-2 is present in semen, is it intermittent or persistent? Moreover, what are the influential factors? (ii) To reveal the nature of the impact of SARS-CoV-2 on offspring, it is necessary to establish an effective animal model to further clarify: how do viruses integrate into human sperm? And how does viral self-replication occur in early embryos?

## Data Availability Statement

The original contributions presented in the study are included in the article/**Supplementary Material**. Further inquiries can be directed to the corresponding author.

## Author Contributions

YH designed the study, reviewed literature, and drafted the manuscript. JW and JC retrieved and summarized the literature. JR and YZ advised on the review and reviewed the final manuscript. XC collected and analyzed the relevant data for the article when the manuscript was revised. All authors contributed to the article and approved the submitted version.

## Conflict of Interest

The authors declare that the research was conducted in the absence of any commercial or financial relationships that could be construed as a potential conflict of interest.

## Abbreviations

COVID-19, Coronavirus Disease 2019; SARS-CoV-2, Severe acute respiratory syndrome coronavirus 2; CT, Chest computed tomography; ACE2, Angiotensin-converting enzyme II; EPS, expressed prostatic secretion; PR, Progressive spermatozoa; NP, Non-progressive; IL-6, Interleukin-6; TNF-α, Tumor necrosis factor-α; MCP-1, Monocyte chemotactic protein-1; RT-PCR, Real-time polymerase chain reaction; DFI, DNA fragmentation index; H&E, Hematoxylin and eosin; TEM, Transmission electron microscopy; BTB, Blood-testis barrier; AT1R, Angiotensin II type 1 receptor; AT2R, Angiotensin II type 2 receptor; PI3K/AKT, Phosphoinositide 3-kinase and protein kinase B; TMPRSS2, Transmembrane serine protease 2; OS, Oxidative stress; T, Testosterone; LH, Luteinizing hormone; FSH, Follicle-stimulating hormone.
