# Supplementary material for: Effect of COVID-19 on Male Reproductive System – A Systematic Review
Source: Front Endocrinol (Lausanne). 2021 May 27;12:677701. doi: 10.3389/fendo.2021.677701 (PMC8190708; doi:10.3389/fendo.2021.677701)
Supplement: Supplementary file 1 [file Presentation_1.pdf]

Search Strategy

We used a broad inclusive search strategy so as not to miss a seminal contribution. The comprehensive search was conducted by an experienced information specialist.

The literature search in PubMed, CBMDisc, China National Knowledge Infrastructure (CNKI), and Wanfang Database used the following search terms:

(severe acute respiratory syndrome coronavirus 2 [All Fields] OR SARS-CoV-2 [All Fields] OR 2019-ncov [All Fields] OR coronavirus disease 2019 [All Fields] OR COVID-19 [All Fields]) AND (semen [All Fields] OR sperm [All Fields] OR seminal [All Fields] OR spermatic fluid [All Fields] OR seminal fluid [All Fields] OR testis [All Fields] OR testes [All Fields] OR testicular [All Fields] OR prostatic secretion [All Fields] OR prostatic fluid [All Fields] OR male reproductive tract [All Fields] OR male genital tract [All Fields] OR male reproductive system [All Fields] OR male gonadal function [All Fields] OR offspring [All Fields] OR Paternal-child transmission [All Fields] OR Father-to-child vertical transmission [All Fields]).

Our search strategy for Embase and MEDLINE was:

1 'severe acute respiratory syndrome coronavirus 2' OR 'SARS-CoV-2'.

2 '2019 new coronavirus' OR '2019-ncov' OR 'coronavirus disease 2019' OR 'COVID-19'.

3 'semen' OR 'sperm' OR 'seminal' OR 'spermatic fluid' OR 'seminal fluid' OR 'testis' OR 'testes' OR 'testicular' OR 'epididymis' OR 'spermatic cord' OR 'prostatic secretion' OR 'prostatic fluid'.

4 'male reproductive tract' OR 'male genital tract' OR 'male reproductive system' OR 'male fertility' OR 'offspring' OR 'Paternal-child transmission' OR 'Paternal-neonatal transmission' OR 'Father-to-child vertical transmission'.

5 1 OR 2.

6 5 AND 3.

7 5 AND 4.

8 6 OR 7

To further ensure a comprehensive search, authors manually scanned the references of the included articles and suitability was determined. Articles were selected if they met any of the following inclusion criteria: conducted in human, identification of SARS-CoV-2 in semen, discussion of SARS-CoV-2 in semen, discussion of SARS-CoV-2 in testis, discussion of father-to-child transmission, the inheritance of offspring, and male reproduction. This study was registered with the PROSPERO on 17 February 2021 (https://www.crd.york.ac.uk/PROSPERO/#joinuppage).
